# Extrapancreatic Autoantibody Profiles in Type I Diabetes

**DOI:** 10.1371/journal.pone.0045216

**Published:** 2012-09-21

**Authors:** Peter D. Burbelo, Evan E. Lebovitz, Kathleen E. Bren, Ahmad Bayat, Scott Paviol, Janet M. Wenzlau, Katherine J. Barriga, Marian Rewers, David M. Harlan, Michael J. Iadarola

**Affiliations:** 1 Neurobiology and Pain Therapeutics Section, Laboratory of Sensory Biology, and National Institute of Dental and Craniofacial Research, National Institutes of Health, Bethesda, Maryland, United States of America; 2 Diabetes Center for Excellence, University of Massachusetts Medical School, Worcester, Massachusetts, United States of America; 3 Barbara Davis Center for Childhood Diabetes, University of Colorado Denver, Aurora, Colorado, United States of America; University of Siena, Italy

## Abstract

Type I diabetes (T1D) is an autoimmune disease characterized by destruction of insulin-producing β-cells in the pancreas. Although several islet cell autoantigens are known, the breadth and spectrum of autoantibody targets has not been fully explored. Here the luciferase immunoprecipitation systems (LIPS) antibody profiling technology was used to study islet and other organ-specific autoantibody responses in parallel. Examination of an initial cohort of 93 controls and 50 T1D subjects revealed that 16% of the diabetic subjects showed anti-gastric ATPase autoantibodies which did not correlate with autoantibodies against GAD65, IA2, or IA2-β. A more detailed study of a second cohort with 18 potential autoantibody targets revealed marked heterogeneity in autoantibody responses against islet cell autoantigens including two polymorphic variants of ZnT8. A subset of T1D subjects exhibited autoantibodies against several organ-specific targets including gastric ATPase (11%), thyroid peroxidase (14%), and anti-IgA autoantibodies against tissue transglutaminase (12%). Although a few T1D subjects showed autoantibodies against a lung-associated protein KCNRG (6%) and S100-β (8%), no statistically significant autoantibodies were detected against several cytokines. Analysis of the overall autoantibody profiles using a heatmap revealed two major subgroups of approximately similar numbers, consisting of T1D subjects with and without organ-specific autoantibodies. Within the organ-specific subgroup, there was minimal overlap among anti-gastric ATPase, anti-thyroid peroxidase, and anti-transglutaminase seropositivity, and these autoantibodies did not correlate with islet cell autoantibodies. Examination of a third cohort, comprising prospectively collected longitudinal samples from high-risk individuals, revealed that anti-gastric ATPase autoantibodies were present in several individuals prior to detection of islet autoantibodies and before clinical onset of T1D. Taken together, these results suggest that autoantibody portraits derived from islet and organ-specific targets will likely be useful for enhancing the clinical management of T1D.

## Introduction

Type 1 diabetes (T1D) involves T cell-mediated attack on β-islet cells in the pancreas resulting in a loss of insulin production [1]. The T cell antigen specificity appears to be reflected in the B-cell repertoire as indicated by the presence of circulating autoantibodies against islet cell autoantigens such as insulin, GAD65, IA-2, IA-2β, and/or ZnT8 [2], [3]. Detection of these autoantibodies can aid in the diagnosis of T1D and in distinguishing different diabetes subtypes including latent autoimmune diabetes of adults (LADA), maturity onset diabetes of the young (MODY), and type 2 diabetes (T2D) [2], [3]. In addition to being useful for diagnosis, anti-islet autoantibodies usually appear years before the onset of T1D and can be used for prediction and potential prevention studies [2], [3]. However, these autoantibody tests are not completely specific in that many children from high risk families fail to develop diabetes, but show transient or sustained islet antibodies. The most reliable factors for identifying future onset of T1D include autoantibody seropositivity against multiple islet antigens, high antibody titers, young age at seroconversion, and persistent autoantibodies against insulin [4], [5], [6], [7], [8], [9], [10], [11].

In T1D, the detection of autoantibodies against organ-specific targets can also be used clinically for identifying co-occurring autoimmune conditions such as thyroiditis, Addison's disease, celiac disease, and autoimmune gastritis [12], [13], [14], [15], [16], [17], [18]. For example, autoimmune thyroiditis is common in T1D patients (15%–30%) and is associated with anti-thyroid peroxidase (TPO) and/or thyroglobulin autoantibodies [14], [15], [16], [19], [20], [21]. Clinical and subclinical forms of celiac disease are also found in T1D and can be identified serologically by anti-IgA autoantibodies against tissue transglutaminase (TGM) [17], [18], [19], [22]. T1D subjects often show celiac disease (10–12%) compared to 0.5% prevalence in the general population. Autoimmune gastritis, accompanied by autoantibodies directed against the gastric parietal cells, is also common in T1D (16%) and can cause iron deficiency anemia and pernicious anemia and can result in a higher risk of gastric cancer [13], [23], [24]. Other less prevalent autoimmune conditions have also been described in T1D patients including Addison's disease associated with anti-21-hydroxylase autoantibodies [19], [22]. A recent large survey found that 33% of T1D children had autoantibodies against TPO, TGM, and/or 21-hydroxylase [22]. Further investigation of incidence and overlap of different types of organ-specific autoantibodies in T1D and their relationship with autoantibodies to GAD65, IA-2, IA-2β, and ZnT8 autoantibodies is clearly warranted.

The detection of autoantibodies against both islet cell and organ-specific targets requires highly sensitive and specific immunoassays such as the liquid phase radiobinding assay (RBA) because solid phase immunoassays miss many diagnostically useful conformational epitopes [25], [26]. As an alternative to RBA, luciferase immunoprecipitation systems (LIPS), which utilizes light-producing recombinant autoantigens, has been used to efficiently detect autoantibodies associated with a variety of human conditions [27], [28]. Side-by-side comparisons have shown that LIPS and RBA possess similar diagnostic sensitivity for detecting autoantibodies against several islet cell autoantigens including IA2, IA2-β, and GAD-65 autoantibodies in T1D [27], [28]. However, compared to RBA, LIPS contains several advantages in that it does not require radioactivity, requires only a relatively small amount of serum (1 µl) and has a wider dynamic range of antibody detection [25], [27], [28]. In this study, LIPS was used to investigate both islet and organ-specific immunoreactivity against a panel of autoantigens to better understand the autoantibody profiles in T1D.

## Materials and Methods

### Ethics Statement

Informed written consent was obtained from all subjects in accordance with the human experimentation guidelines of the Department of Health and Human Services and the studies were conducted according to the principles expressed in the Declaration of Helsinki.

Serum samples from the 2009 and 2010 Diabetes Autoantibody Standardization Program (DASP) cohorts containing T1D patients and healthy volunteers were obtained from Dr. Patricia Mueller at the Center for Disease Control. All laboratories contributing sera for the DASP cohorts were required to have institutional review board approval for human research. All DASP samples were analyzed anonymously as de-identified blinded samples. The Diabetes Autoimmunity Study in the Young (DAISY) was approved by Colorado Multiple Institutional Review Board (COMIRB) for the University of Colorado Denver and a consortium of health care facilities in Colorado [6]. For the DAISY samples, written informed consent was obtained from the parents of each study subject at genetic screening and again at enrollment in the DAISY follow-up.

### Study Population

Serum samples from the DASP 2009 and DASP 2010 cohorts were tested as blinded samples. Antibody titers were determined before unblinding. The DASP 2009 samples consisted of 93 controls and 50 T1D subjects, while the DASP 2010 samples contained 90 controls and 50 T1D subjects. As part of these DASP studies, clinical information such as age, gender, and other patient details are unavailable for analysis.

Prospectively collected samples from children at a high risk from first degree relatives with T1D were obtained from Diabetes Autoimmunity Study in the Young [6]. Additional covariants such as HLA genotype, autoantibody seropositivity, and age of onset were also available for analysis for these 75 subjects.

### LIPS antigens

LIPS tests for three known diabetes autoantigens, IA2, GAD65, and IA2-β, have previously been described [27], [28]. A new plasmid for generating a *Renilla* luciferase (Ruc) antigen fusion of ZnT8 [29] was constructed using the C-terminal intracellular region corresponding to (amino acids 268–369) of GenBank accession NP_776250.2 essentially as previously described [28]. In addition to the ZnT8-325R isoform, site-directed mutagenesis was further used to generate two additional major polymorphic isoforms, ZnT8-325Q and ZnT8-325W, and DNA sequencing was performed to ensure their integrity.

LIPS tests for detecting organ-specific autoantibody targets included the gastric ATPase (ATP4B), thyroid peroxidase (TPO), aquaporin-4 (AQP-4), glial fibrillary acidic protein (GFAP), and KCNRG have been previously described [30], [31]. Of particular interest, the Ruc-ATPB4 construct was generated using amino acids 1–238 of GenBank accession NP_000696.1. Two additional targets, TGM and S100-β, were generated as Ruc-antigen fusions; TGM corresponded to GenBank accession NP_945189 (amino acids 2–548) and S100-β corresponded to GenBank accession NP_006263 (amino acids 2–92). Other LIPS tests for selected cytokines including interferon-α (INF-α), interferon-γ (INF-γ), interleukin-1α (IL-1α), interleukin-6 (IL-6), and the interleukin-12 p35 (IL-12 p35) subunit have also previously been described [32].

### LIPS testing

For LIPS testing of the two different DASP cohorts of serum samples, master plates were constructed by diluting serum 1/10 in buffer A (50 mM Tris, pH 7.5, 100 mM NaCl, 5 mM MgCl_2_, 1% Triton X-100 and 0.001% bromophenol red), in a deep 96-well microtiter plate and mixed thoroughly [33]. LIPS testing was preformed essentially as described. For detecting anti-TGM IgA autoantibodies, goat anti-human IgA-agarose conjugated beads (Sigma) were substituted for protein A/G beads. Luciferase activity captured on the filter plates was then measured in a Berthold LB 960 Centro microplate luminometer (Berthold Technologies, Bad Wilbad, Germany) using coelenterazine substrate mix (Promega, Madison, WI). All data represent raw antibody titers without subtracting the buffer blanks.

### Statistical analysis

Analysis of the autoantibody titer data was performed with the statistical GraphPad Prism software (San Diego, CA). The results for quantitative antibody levels are reported as the mean titer ±95% confidence interval (CI). Statistical significance was determined using the Mann Whitney *U* test. Cutoffs for sensitivity and specificity were determined based on the mean plus 3 standard deviations (SD) of the control samples. In some cases, receiver operator characteristics (ROC) analysis was used to calculate of sensitivity based on 98% specificity. Antibody values described in the text were rounded to three significant figures.

To analyze the T1D samples from the second DASP cohort for potential autoantibody subgroups, the autoantibody titer values were converted to *Z* scores and a heatmap was created. For this data transformation, the mean and standard deviation for each autoantibody titer from the control samples were calculated. Of note, one control outlier sample in the DASP 2010 cohort, with extremely high anti-IL-6 autoantibodies, which was also positive for GAD65 and S100-β autoantibodies, was excluded from the analysis for calculating the average and standard deviations of the control samples. The values of the T1D samples were then color-coded to signify relevant antigen-antibody seropositive responses that were greater than the mean plus 3 SD of the 89 control subjects. For the heatmap, a jelly-bean color palette ranging from green to black was used to indicate low and high titers, respectively, and was used to signify the relative Z-score compared to the control non-diabetic samples.

## Results

### Robust detection of autoantibodies against islet antigens and the gastric ATPase in T1D

**Figure 1:**
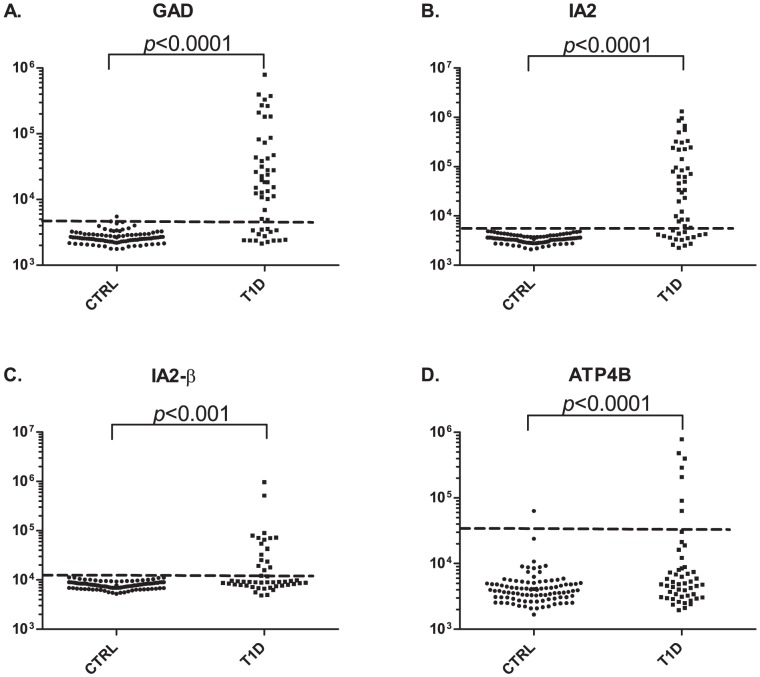
Autoantibodies in T1D and control subjects from DASP 2009. The mean antibody titer and 95% confidence interval for (**A**) GAD65 (**B**) IA2, (**C**) IA2-β, and (**D**) ATP4B titers in the 89 controls and 50 T1D subjects were plotted on the Y-axis using a log_10_ scale. Each symbol represents a sample from one individual. The dashed line represents the cut-off level for determining seropositivity and is derived from the mean plus 3 standard deviations of the antibody titer of the controls. *P* values for the different groups were calculated using the Mann Whitney *U* test.

The quantitative LIPS assay was used to evaluate autoantibodies against anti-islet cell targets IA2, IA2-β and GAD65 and an organ-specific target, ATP4B, representing the small subunit of the gastric ATPase. For these studies, blinded serum samples from the 2009 DASP cohort representing control and T1D samples taken at the time of diabetes diagnosis were used. Following unblinding, dramatic differences in antibody titers were often found between controls (n = 93) and T1D subjects (n = 50). For example, the mean anti-GAD65 antibody titer in the controls was 2,700 LU (95% CI; 2,570–2,840) and was much lower than the T1D samples with a mean titer of 75,300 LU (95% CI; 34,100–117,000) (Figure 1A). The autoantibody titers for GAD65 ranged from 1,760 to 788,000 LU. Similarly, the anti-IA2 antibody titers also showed a wide dynamic range of detection from 2,100 to 1,320,000 LU. The anti-IA2 antibody mean titer in the controls was 3,500 LU (95% CI; 3,320–3,590) and was much lower than the T1D samples with a mean of 115,000 LU (95% CI; 76,300–235,000) (Figure 1B). Autoantibodies against IA2-β autoantibodies also showed marked differences between the controls and the T1D samples (Figure 1C), but were not as informative as IA2. Applying cut-off values based on the mean plus 3 standard deviations of the controls, GAD65, IA2, and IA2-β autoantibodies demonstrated 67% and 98.9% specificity, 77% sensitivity and 100% specificity, and 60% sensitivity and 100% specificity for the diagnosis of T1D, respectively.

Testing for anti-gastric autoantibody titers revealed titers ranging from 1,670 to 782,000 LU in the DASP samples (Figure 1D). Based on a cut-off corresponding to the mean plus 3 standard deviations of the control subjects, 7 of the 50 T1D samples (14%) were seropositive above the cut-off for anti-ATP4B antibodies and showed much higher titers with 96% specificity. ROC analysis also revealed that at 98% specificity, 16% of the T1D were seropositive for anti-gastric ATPase autoantibodies. Interestingly, one of the ATP4B seropositive T1D subjects was negative for anti-GAD65, anti-IA2, and anti-IA2-β autoantibodies. Additional analysis revealed that individual T1D autoantibody profiles against these four autoantigens were highly variable in titer and in the spectrum of immunoreactivity (data not shown).

### Evaluation of autoantibodies against islet, organ-specific and other autoantigens in T1D

Based on our initial results with the DASP 2009 cohort demonstrating marked heterogeneity against three β-cell targets and ATP4B, autoantibodies against a more extensive panel of islet, organ-specific, and other potential autoantigens was determined in a second, independent sample set representing the coded DASP 2010 cohort. For these tests, 6 different β-cell targets were examined including GAD65, IA2, IA2-β and 3 different ZnT8 subtypes [34]. Based on the literature, autoantibodies were also tested against six potential organ–specific antigens implicated in T1D and/or other autoimmune conditions including ATP4B, TGM2, TPO, KCNRG, AQP-4, and GFAP. Lastly, autoantibodies were examined against S100-β and five cytokines including INF-α, INF-γ, IL-6, IL-1α and IL-12 p35. For LIPS analysis of these eighteen targets, all autoantibody titers were determined first with masked samples prior to unblinding.

As shown in Figure S1, analysis of anti-GAD65, anti-IA2, and anti-IA-2β in the 90 controls and 50 T1D from the 2010 DASP samples showed a similar distribution of autoantibody titers as seen in the DASP 2009 cohort. In these 2010 DASP samples, the diagnostic performance of autoantibodies against GAD65, IA2, and IA2-β demonstrated 68%, 70%, and 46% sensitivity, respectively (Figure S1). From evaluating autoantibodies against three polymorphic isoforms of ZnT8, the most common seropositivity was observed with the ZnT8-R isoform (Figure S2). There was a wide dynamic range of autoantibody titers observed against ZnT8-R in T1D patients, ranging from 1,930 LU to 187,000 LU and applying the standard cut-off based of the mean plus 3 SD showed 32% sensitivity with 100% specificity (Fig. S2). Autoantibodies against the ZnT8-W isoform also showed a similar dynamic range of detection and demonstrated 24% sensitivity and 100% specificity (Figure S2). In contrast, examination of autoantibodies against ZnT8-Q isoform did not detect any statically significant autoantibody responses in the T1D subjects compared to the controls (data not shown). The lack of immunoreactivity against this version may reflect the poor folding of Ruc-ZnT8-Q isoform. Nevertheless, combining the diagnostic results of autoantibodies to either ZnT8-R or ZnT8-W, 42% of the T1D subjects were seropositive for anti-ZnT8 autoantibodies (with 100% specificity).

### Organ specific and other Autoantibodies in the DASP 2010 Cohort

**Figure 2:**
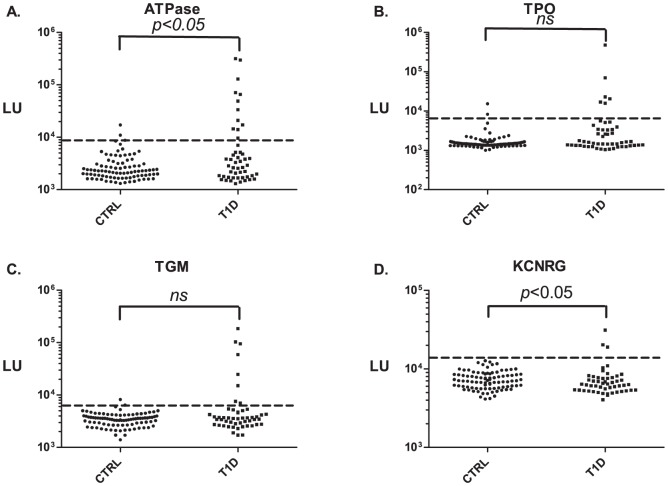
Profiling organ specific autoantibodies in a second cohort of T1D and control subjects. Autoantibodies were evaluated in the DASP 2010 samples containing 50 T1D patients and 90 controls. The autoantibody titers for (**A**) ATP4B, (**B**) TPO, (**C**) TGM, and (**D**) KCNRG, are plotted on the Y-axis using a log_10_ scale. Each symbol in the scatterplots represents serum from one subject. The dashed line represents the cut-off level used for determining seropositivity and is derived from the mean plus 3 SD of the antibody titer of the 90 controls.

From profiling different organ-specific and cytokine targets and using the value of the mean plus 3 SD of the controls as a cut-off for each antigen, autoantibodies against ATP4B were the most common. Similar to the previous cohort, 24% (12/50) of the T1D subjects were seropositive for ATP4B with 98% (88/90) specificity (Figure 2A). Anti-TPO antibodies were seropositive in 6 of 50 (12%) T1D subjects, but two controls were seropositive and just above the cut-off (Figure 2B). Eight T1D subjects showed anti-IgA seropositivity against TGM (16% sensitivity with 99% specificity) (Figure 2C). In the case of KCNRG, only 3 T1D subjects were seropositive with 100% specificity (Figure 2D). Analysis of immunoreactivity against AQP-4, GFAP, and five cytokines (interferon-α, interferon-γ, interleukin-6, interleukin-1α, and IL-12 p35) revealed that none of these proteins showed statistically relevant autoantibody responses in the T1D subjects compared to the controls (Figure S3). However, five T1D patients showed statistically significant autoantibody titers above the cut-off against S100-β but 3 controls also were seropositivity and again just above the cut-off (Figure S3). Additional ROC analysis also revealed that at 98% specificity, 8% of the T1D subjects were seropositive for anti-S100-β autoantibodies.

### Heatmap analysis identifies two major autoantibody subgroups in T1D

**Figure 3:**
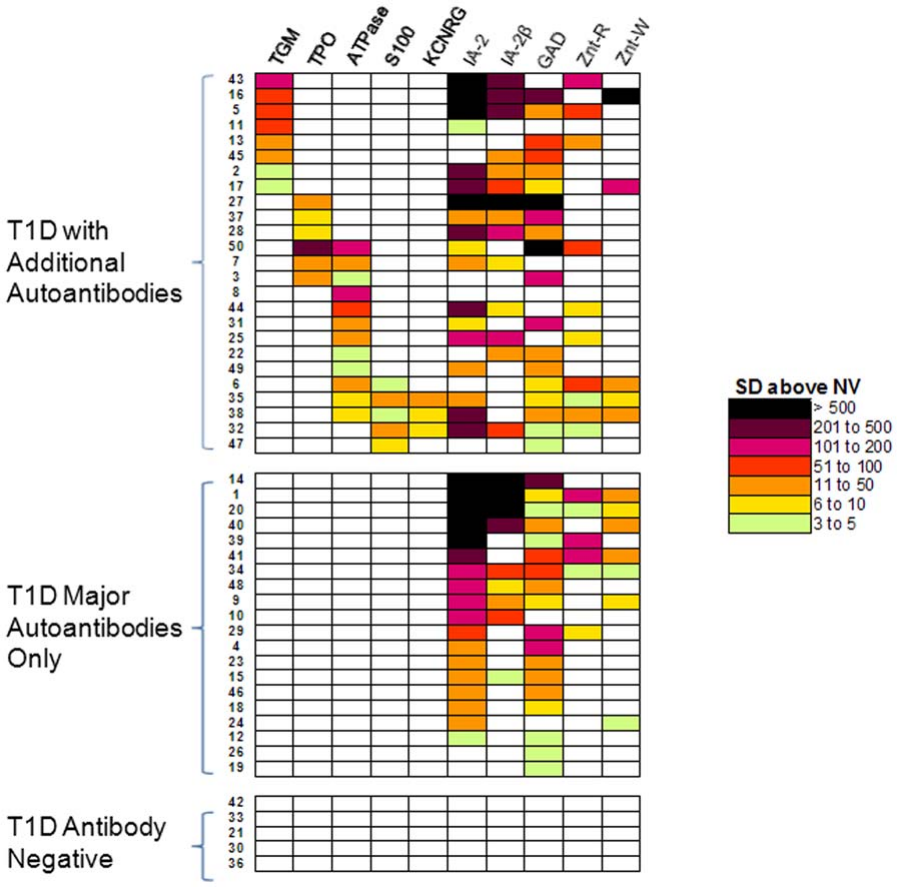
Autoantibody portraits in T1D subjects. Individual autoantibody titers in the 50 T1D subjects from the DASP 2010 cohort are presented as a heatmap. As described in the material and methods, color code reflects the relative titers in standard deviations above the mean plus three SD of the control subjects for each of the 10 antibody-antigen pairs. Individual antibody profiles were then manually assembled based on the presence or absence of particular organ-specific autoantibodies and then by autoantibodies to the major islet autoantigens.

To understand the individual immunoreactivity against this panel of autoantigens, a data-driven, statistical approach and heatmap analysis was used. As described in the material and methods, antibody titer data in the T1D subjects from the DASP 2010 were first transformed via *Z*-score statistic using the non-diabetic control subjects as reference. Using these values, a color-coded heatmap was constructed, in which single row represents the autoantibody portrait in a given T1D subject (Figure 3). Individual antibody profiles were then manually assembled based on the presence or absence of particular organ-specific autoantibodies and then on autoantibodies to the major islet autoantigens. Analysis of the heatmap revealed three autoantibody subgroups (Figure 3). The islet only group (40%; 20/50) contained subjects who had autoantibodies only to islet cell autoantigens, while the second group (50%; 25/50) contained subjects who had autoantibodies against at least one non-islet target. A few diabetic subjects (10%; 5/50) showed no statistically significant autoantibodies above the control cut-offs to any of the antigens tested. Analysis of the two major groups showed that the individual T1D autoantibody portraits were highly variable in titer and in the spectrum of immunoreactivity. For example, some diabetic subjects showed no immunoreactivity to any of the 10 autoantigens, while other individuals showed immunoreactivity to a maximum of 7 autoantigens. Excluding the IA2-β seropositive samples that were almost exclusively detected in IA2 seropositive samples, the autoantibodies against islet and/or non-islet targets poorly correlated with each other (Figure 3). Further analysis of the ZnT8 autoantibodies in the heatmap highlighted the likely polymorphism-dependent specificity of these autoantibodies, in which 14 subjects reacted only with either the ZnT8-R or ZnT8-W isoform, while only 7 subjects were positive for both isoforms.

Detailed inspection of immunoreactivity against non-islet targets revealed that anti-gastric ATP4B autoantibodies were the most prevalent (24%; 12/50). Of the 12 seropositive T1D subjects, 6 subjects showed organ-specific immunoreactivity only to the ATP4B. Interestingly, one of the individuals showed sole immunoreactivity against the gastric ATPase and no other islet or non-islet autoantibodies. Nevertheless, the other half of the ATP4B seropositive samples were positive for either anti-TPO or anti-S100-β autoantibodies (Figure 3). From the 6 subjects with statistically significant anti-TPO autoantibodies, 3 individuals were also seropositive for anti-ATP4B autoantibodies, and the three others were only seropositive for islet cell autoantibodies. Anti-TGM autoantibodies were also common in the cohort, but not a single anti-TGM seropositive individual was co-positive for autoantibodies against other non-islet antigens (Fig. 3). Finally, the 5 subjects seropositive for anti-S100-β were found in the most immunoreactive subjects and three were also seropositive for KCNRG autoantibodies. Taken together these results highlight the complexity of diverse organ specific autoantibodies present in the T1D subjects and support the development of individual autoantibody portraits for enhancing the diagnosis and clinical management of T1D.

### Detection of gastric ATPase autoantibodies before the onset of T1D

**Figure 4:**
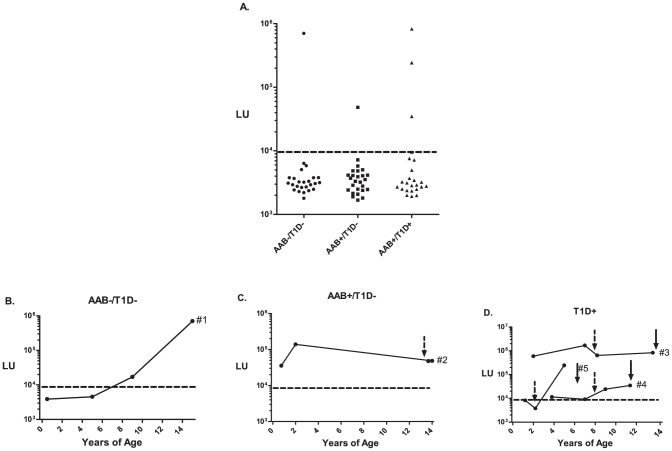
Predictive value of anti-gastric autoantibodies in high risk children. (**A**) Anti-ATP4B autoantibodies were evaluated in pediatric serum samples (n = 75) from the high risk DAISY cohort containing three clinically defined subgroups: islet autoantibody negative and T1D negative (AAB−/T1D−), islet autoantibody positive but currently without T1D (AAB+/T1D), and islet autoantibody positive and diagnosed with T1D (AAB+/T1D+). Only the last available longitudinal serum sample from these children was initially evaluated. The autoantibody titers are plotted on the Y-axis using a log_10_ scale. The dashed line represents the previous cut-off level for determining seropositivity. (**B**) The five seropositive children were further analyzed for anti-ATP4B autoantibodies using available longitudinal serum samples. The titer value shown for the last serum sample was taken from the results obtained in panel A. The stippled arrow represents the age of first detectable islet autoantibodies and the solid arrow marks the time of T1D onset. The dashed line represents the cut-off for determining seropositivity and was represents the ATP4B cut-off value from the DASP 2010 samples.

The frequency and onset of gastric ATPase autoantibodies were also examined in a third cohort from the DAISY comprising prospectively collected longitudinal serum samples from children at risk of T1D. These 75 samples were from 3 clinically defined subgroups: 25 pediatric samples from non-T1D individuals negative for islet autoantibodies, 26 pediatric samples seropositive for islet autoantibodies but currently without T1D, and 24 T1D pediatric samples positive for islet autoantibodies that developed T1D. As an initial screen, only the last available serum sample from each of the 75 high-risk children was evaluated. Analysis of ATP4B autoantibodies revealed that five of the high risk children samples were seropositive including one child who was negative for islet autoantibodies and did not develop T1D, one child who was positive for islet cell autoantibodies and also did not develop T1D and three T1D children (3/24; 15%) who were also co-positive for islet cell autoantibodies (Figure 4A).

To gain insight into whether gastric autoantibodies might be present before the onset of T1D and to understand the development of these autoantibodies over time in the high risk children without T1D, the additional available longitudinal samples from these five seropositive children were evaluated. In the case of the child (#1) negative for islet autoantibodies and without T1D, ATP4B seropositivity was first observed at age 9 and thereafter the antibody titers increased until the last time point at age 15 (Figure 4B and Table S1). The single child (#2) with islet autoantibodies but currently without T1D, showed anti-gastric ATPase autoantibodies at the 8 month earliest time point and these antibodies occurred prior to the appearance of the anti-insulin autoantibodies (Figure 4C and Table S1). From the three children who developed T1D and were seropositive for ATPB4, two of the subjects (#3 and #4) had anti-gastric ATP4B autoantibodies earlier than anti-islet autoantibodies and before the subsequent development of T1D (Figure 4D and Table S1). For example, in subject #3, very high titers of anti-gastric ATP4B autoantibodies (i.e. 590,100 LU) were present at age 2 and these antibodies were detectable much earlier than anti-islet autoantibodies that arose at almost age 8 or subsequent development of T1D at age 13 (Figure 4D). In the remaining T1D subject (#5), the anti-gastric autoantibody titers appeared at age 5, which was after the appearance of anti-islet antibodies that occurred at age 2 but before T1D onset at age 6 (Figure 4D and Table S1).

## Discussion

Our work with the LIPS autoantibody profiling technology provides several novel insights for understanding T1D. Analysis of the statistically significant autoantibody responses at the time of diagnosis revealed two major autoantibody subgroups of T1D patients. One subgroup represented diabetic subjects who demonstrated only detectable islet cell autoantibodies, while the other subgroup showed a more complicated picture with both islet cell and non-islet cell autoantibodies present. A previous survey of three organ–specific autoantigens in 491 T1D children revealed a 33% frequency [22], however our finding of 50% with organ-specific autoantibodies at the time of diagnosis suggests that these autoantibodies are more prevalent than previously appreciated and likely represent both clinical and sub-clinical phenotypes of celiac disease, autoimmune thyroid disease, and gastritis [35]. The overall higher incidence of organ-specific autoimmunity of approximately 50% detected in our study was mainly due to the inclusion of gastric ATPase autoantibodies which were not previously tested in parallel with other organ-specific autoantibodies. The prevalence of anti-gastric ATPase autoantibodies in approximately 16–24% of T1D subjects in the two different DASP cohorts is also consistent with other published findings that used immunofluorescence [13], [24] and recently developed radioimmunoprecipitation assay [36]. In contrast to previous studies [24], [37], no significant association of anti-gastric autoantibodies was detected with autoantibodies against GAD65 or TPO in our cohort, but this lack of correlation in our study may be due to our small sample size. Autoantibodies against a potential lung autoantigen, KCNRG, were also detected in three diabetic subjects. Previously, anti-KCNRG autoantibodies were identified as a biomarker of pulmonary disease in autoimmune polyendocrine syndrome type I patients, a condition with a high co-occurrence of T1D [38]. If autoantibodies against this lung protein are confirmed, these results would further the idea that T1D is a disease with diverse humoral immunoreactivity outside the pancreatic β-islet cells.

Although two major autoantibody subgroups were observed in T1D, each individual had a remarkably unique breadth and spectrum of autoantibody responses. The individual complexity of the autoantibody profile against the 10-antigen panel is due in part to the lack of correlation between any two autoantibody targets. Additionally, the presence of organ-specific autoantibodies was not predictable based on the spectrum or titers against islet autoantigens. It is also important to point out that several proposed autoantigen targets in T1D and in other autoimmune conditions, including the astrocytic protein GFAP [39] and a number of cytokines, failed to show statistically significant immunoreactivity in T1D. Of note, based on the low sensitivity of the LIPS ZnT8 test compared to published studies with RBA [40], the ZnT-8 autoantigen folding as a Ruc fusion may not be optimum for the detection of the many conformational epitopes and further testing of other constructs are needed to achieve better diagnostic performance.

The prediction of the future onset of T1D in high risk children relies in part on the detection of autoantibodies against insulin, GAD65, IA-2, IA-2β, and/or ZnT8, in which increased risk of developing T1D is associated with the presence of multiple autoantibodies [6], [10], [11]. From evaluating samples from the DAISY cohort for autoantibodies against ATP4B, 12% (3/24) of the T1D children showed anti-gastric autoantibodies years before they developed T1D. The observation of two additional children from high risk families with seropositive anti-gastric ATPase autoantibodies who did not develop diabetes is also consistent with the fact that the gastric autoantibodies are not completely specific for T1D and may reflect the high incidence of extrapancreatic autoimmune manifestations in non-diabetic children of diabetic parents [21]. Nevertheless, the identification of anti-gastric autoantibodies in some children before the detection of anti-islet autoantibodies highlights the extrapancreatic humoral immune dysfunction present at an early age (<4 years of age) in these children. Together with our findings that 50% of T1D subjects show organ specific autoantibodies suggests the possibility that other organ-specific autoantibody responses (e.g. TPO, TGM) may also be present before T1D onset. Future studies detecting islet and organ-specific autoantibodies in parallel with antibodies against infectious agents (e.g. enteroviruses) in prospective high risk children may provide additional insights into the potential environmental triggers of these co-occurring autoimmune conditions.

## Supporting Information

Figure S1 **Autoantibodies in T1D and control subjects from DASP 2010.** The mean antibody titer and 95% confidence interval for (**A**) GAD65, (**B**) IA2, and (**C**) IA2-β in the 90 controls and 50 T1D subjects were plotted on the Y-axis using a log_10_ scale. Each symbol represents a sample from one individual. The mean is shown by the short line with 95% CI error bars. The dashed line represents the cut-off level for determining seropositivity and is derived from the mean plus 3 standard deviations of the antibody titer of the controls. *P* values for the different groups were calculated using the Mann Whitney *U* test.
(EPS)

Figure S2 **Autoantibodies against ZnT8 in the DASP 2010 samples.** Autoantibodies against ZnT8-R and ZnT8-W were evaluated in the DASP 2010 samples containing 50 T1D patients and 90 controls. Each symbol in the scatterplots represents serum from one subject and the autoantibody titers for are plotted on the Y-axis using a log_10_ scale. The dashed lines represent the cut-off levels used for determining seropositivity and are derived from the mean plus 3 SD of the antibody titer of the 90 controls.
(EPS)

Figure S3 **Antibody profiling candidate autoantigens in T1D and control subjects.** Autoantibodies were evaluated in the DASP 2010 samples containing 50 T1D patients and 90 controls. The autoantibody titers for (A) GFAP, (B) AQP-4, (C) Interferon-γ, (D) Interleukin-6, (E) Interleukin 1-α, (F) Interferon-α, (G) Interleukin-12 p35 and (H) S100-β are plotted on the Y-axis using a log_10_ scale. Each symbol in the scatterplots represents serum from one subject. The dashed line represents the cut-off level used for determining seropositivity and is derived from the mean plus 3 SD of the antibody titer of the 90 controls.
(EPS)
